# Management of Foot Ulcers and Chronic Wounds with Amniotic Membrane in Comorbid Patients: A Successful Experience

**DOI:** 10.3390/biomedicines12102380

**Published:** 2024-10-18

**Authors:** Mónica Rodríguez-Valiente, Ana M. García-Hernández, Cristina Fuente-Mora, Javier Sánchez-Gálvez, Eva María García-Vizcaino, Elena Tristante Barrenechea, Gregorio Castellanos Escrig, Sergio David Liarte Lastra, Francisco Jose Nicolás

**Affiliations:** 1 Department of General Surgery, Hospital Clínico Universitario Virgen de la Arrixaca, Biomedical Research Institute of Murcia (IMIB-Arrixaca), El Palmar, 30120 Murcia, Spain; grcase@gregoriocastellanosescrig.com; 2 Faculty of Nursing, UCAM Catholic University of Murcia, Av. Jerónimos 135, Guadalupe, 30107 Murcia, Spain; javiersanchez@ucam.edu (J.S.-G.); emgarcia@ucam.edu (E.M.G.-V.); 3 Research Group on Molecular and Cellular Biology Solutions in Regenerative Medicine, UCAM Catholic University of Murcia, Av. Jerónimos 135, Guadalupe, 30107 Murcia, Spain; 4 Cell Therapy Unit at Hospital Clínico Universitario Virgen de la Arrixaca, Biomedical Research Institute of Murcia (IMIB-Arrixaca), El Palmar, 30120 Murcia, Spain; 5 Clinical Trial Plataforms FFIS, Biomedical Research Institute of Murcia (IMIB-Arrixaca), El Palmar, 30120 Murcia, Spain; cristina.fuente@imib.es; 6 Laboratorio de Regeneración, Oncología Molecular y TGF-ß IMIB-Arrixaca, Biomedical Research Institute of Murcia (IMIB-Arrixaca), El Palmar, 30120 Murcia, Spain; franciscoj.nicolas2@carm.es

**Keywords:** amniotic membrane, chronic wounds, cell therapy, quality of life

## Abstract

Chronic wounds are defined as those with disturbances in normal healing. They involve symptoms like exudate, odor, pain or impaired mobility, severely impacting life quality. In the case of patients with additional comorbidities, these are known to aggravate the healing impairment. Amniotic membrane (AM) is gaining attention for its regenerative potential, as it has shown promise in treating hard-to-heal wounds, such as diabetic foot ulcers. This work examines a series of five patients who, while suffering an array of other chronic conditions, were treated with AM for the management of non-healing chronic ulcers. Inclusion criteria involved patients with lesions that have been active at least for six weeks and resistant to multiple treatments, accompanied by complex underlying pathologies affecting cardiovascular, immune or renal functions. Exclusion criteria included untreated active infections and patients undergoing other experimental treatments. The mean age of the patients was 68.4 ± 5.2 years. Wounds were treated once a week with AM, following standardized procedures. The variables measured included pain levels, microorganism presence, wound reduction and the number of AM applications to recovery. The median pain VAS score decreased significantly from seven at the start to two at the end of procedures. Four out of five patients achieved complete epithelialization, while the remaining patient showed significant reductions of 40% in wound size after 14 months. Our results confirm how the application of AM is a safe and effective resource for the management of chronic wounds in patients with serious comorbidities, enhancing patients’ quality of life, firstly by reducing pain, later by allowing recovery. Future research, including molecular analyses of wound exudates before and after AM treatment, can contribute to better understanding and fine tuning of this therapeutic resource.

## 1. Introduction

The skin constitutes a crucial and effective barrier against environmental elements. When skin injury occurs, the body initiates a series of complex events to reestablish this natural protection [1]. Chronic wounds are characterized by their minimal or absent tendency to heal. The most common chronic wounds in the lower extremities include arterial, diabetic, pressure, and venous ulcers; yet certain patients may develop chronic wounds due to other underlying pathologies that impair proper healing [1]. Chronic wounds have a significant socioeconomic impact, as they constitute a risk for disability and require long-term care from skilled professionals [2]. Signs and symptoms such as exudate, odor, pain, and reduced mobility are some of the common characteristics observed in these patients, as demonstrated in various studies [3,4,5,6,7]. In most cases, it is accepted that the quality of life of these patients is reduced due to their wounds.

Ample evidence shows that stem cells found in the amniotic membrane (AM) can differentiate in vitro into various cell types, while also possess biological properties that aid in healing. Additionally, they have been proven to synthesize and release biologically active substances [8,9]. Current advanced therapies, like the use of AM, are proving effective in treating specific conditions that become chronic over time [10]. Numerous studies have demonstrated the effectiveness of this tissue in treating different types of wounds and burns [11,12,13]. In fact, AM application in hard-to-heal wounds, such as diabetic foot ulcers, has shown promising results [14].

Our team’s track record is the demonstration of a success story in translational medicine for the development of AM-based advanced therapies, starting from bench-basic research to validating our work in clinical application, all supported by publications [14,15]. In this paper, we add further evidence to this trend by showing a case series of five patients suffering from non-healing chronic diabetic foot ulcers, in the context of additional systemic ailments, showing how all of them benefited from treatment with AM. The success we recorded from such treatment drives us to propose the use of the AM in patients with complex pathologies and chronic wounds that have remained unhealed for at least six weeks.

## 2. Material and Methods

### 2.1. Donor Selection and Informed Consent

The amniotic membrane was obtained from healthy pregnant women at term who met the criteria of regular prenatal care and who underwent elective caesarean section based on obstetric reasons. All donors were interviewed, their risk for having an infectious disease assessed, and they gave informed written consent prior to tissue donation and blood obtention to perform the standard serological screening, including HIV-1/2 antibodies, HIV-antigen, HIV 1 -RNA, HBsAg, HBc antibodies, HBV-DNA, HCV-antibodies, HCV-RNA, syphilis and HTLV I/II antibodies.

### 2.2. Amniotic Membrane Processing

After the caesarean birth, the gynecologist removes the placenta which is then placed on a side table on top of a sterile cloth. The AM is then dissected in the operating room, using surgical forceps and a scalpel, separating it mechanically from the chorionic membrane, carefully to not damage any blood vessels and by making a superficial cut at the base of the umbilical cord. The dissection is obtained by separating the amnion from the chorion, detaching both tissues that surround the placenta and are attached to each other.

Once dissected, the AM is placed in a sterile solution containing 1000 mL of sterile saline (Grifols, Barcelona, Spain) with antibiotics [soltrim^®^ 48 mg (Almirall, SA Barcelona, Spain), tobramycin^®^ 50 mg (Normon, Madrid, Spain) and vancomycin^®^ 50 mg (Normon, Madrid, Spain)].

In our case, the obstetrics operating room is near the Cell Production Unit (CPU), so the AM was transported at room temperature and then it was preserved at 4 °C until processing and preservation. At this time, the Spanish Agency of Medicines and Medical Devices (AEMPS) classifies cryopreserved AM as a somatic cell therapy medicinal product as, although it constitutes a tissue not being subjected to substantial manipulation, it is not intended to be used for the same essential function(s) in the recipient and the donor. As such, AM dressings are generated as a research drug (MA-GH; PEI number 12-008) under the European Union’s Good Manufacturing Practices.

At the CPU, in a class II (laminar flow) safety cabinet, the AM is washed four times with sterile saline solution (Grifols, Barcelona, Spain) to remove traces of blood and remove debris from the chorion. The AM is then chopped into 8.5 cm × 10 cm pieces and sewed at all four corners of each fragment to a sterile Tulgrasum^®^ vaseline gauze dressing (Bama-Geve, SLU, Barcelona, Spain) using a 3.0 braided silk suture (Lorca-Marin, Murcia, Spain). Each AM fragment is then placed in a cryopreservation bag (Macopharma, Tourcoing, France) where a freezing solution made of human albumin (Grifols, Barcelona, Spain) in TC-199 Medium (Sigma-Aldrich, St. Louis, MI, USA) and 15% dimethylsulfoxide (DMSO; Origen, Austin, TX, USA) is added. Then, AM pieces are frozen at −80 °C until use. Quality controls include microbiological testing of the active substance bank and sterility test of the final product, using Bact-ALERT^®^3D (Biomerieux, Marcy-l’Étoile, France).

Just prior to use, AM is thawed in a water bath at 37 °C and then washed three times with sterile saline solution (Grifols, Barcelona, Spain) and then transported to the clinic in a temperature-controlled container, between 2 and 8 °C. The cryopreserved AM is applied fresh. Cell viability for applied AM was confirmed as previously described [16].

### 2.3. Procedure for AM Application in Wounds

AM applications were performed weekly over a clean wound following standardized cleaning procedures. First, the wound is cleaned with a soapy sponge (Jalsosa SL, Granada, Spain) and saline solution (Labesfal, Besteiros, Portugal) to remove contamination from the surrounding skin, bacteria, and remnants of previous dressings. If the wound has necrotic tissue, it is removed by sharp debridement performed with surgical forceps and scalpel. Before AM application, the wound bed is irrigated with sterile saline solution (Labesfal, Besteiros, Portugal) and if infection was suspected, a sample was taken for microbiological analysis, using a swab soaked in Amies medium and then processed according to the hospital’s wound exudate protocol. Before AM application, the fibrin layer at the ulcer bed is debrided with a size 15 scalpel applying gentle scraping. Once the wound is clean and debrided, application of AM is performed. Under sterile conditions, the AM is removed from container and placed, extending it to ulcer edges using sterile forceps and ensuring contact with the ulcer bed. To finish, the AM is covered with a sterile Tulgrasum^®^ Vaseline dressing (Bama-Geve, SLU, Barcelona, Spain). Additionally, sterile gauze is applied over it and one or more crepe dressings or bandages are applied over these if the wound is located on a limb. Finally, the patient is instructed on how to recognize any signs and symptoms of site infection and how to proceed in the event of any complications.

All the data regarding the application procedure was recorded in the patient’s clinical history: batch and number of AM patch, details and characteristics of the wounds. Also, any unexpected events during treatment were recorded as potential adverse reactions in the patient’s medical history. The total number of AM applications per wound was decided based on the clinical evolution of each patient.

### 2.4. Advanced Therapy Approval and Ethical Aspects

The “Cell Therapy Clean Room” unit at the hospital was approved by the Spanish Agency of Medicines and Medical Devices (AEMPS) in 2021 for the production and application of amniotic membrane (AM) as part of routine clinical practice. This approval ensures that the AM is used under medical indication, with patient consent, and with proper documentation in medical records. The AM is considered authorized for clinical use by AEMPS due to the regulation and authorization of the laboratory that manufactures it.

This study is a case series, with written authorization obtained from patients for participation and image publication. The AM is processed in the clean room unit according to the established standard operating procedures (SOP). This includes traceability and comprehensive serological and microbiological control during extraction, processing, and cryopreservation.

### 2.5. Patient Selection and Monitoring

Cases suitable for selection corresponded to patients with chronic wounds lasting more than six weeks, which were resistant to previous treatments and with concomitant presence of complex underlying pathologies. Suitable cases were discarded on the grounds of the presence of untreated active infections, being treated with other experimental treatments simultaneously and patients who did not accept the treatment.

Variables measured included: the presence of adverse events; pain, assessed using a visual analog scale (VAS) from 1 to 10 before the first application, after the third application, and at the end of the treatment; the types of microorganisms present before the application of the amniotic membrane (AM); the number of AM applications; reduction in wound size; and complete epithelialization. Wound-area pictures were taken before the first AM application and before each subsequent application. Photographic images were captured with a Xiaomi Rednote 13 mobile device from a calibrated distance of 30 cm using a stand. Wound measurements were assessed using photographic image analysis. Clinical signs of infection—such as pain, redness, odor, edema, and increased exudate—were evaluated at each AM application.

### 2.6. Data Analysis

Data were analyzed using descriptive statistics to summarize patient and wound characteristics. Continuous variables, such as age and duration of treatment, were expressed as mean ± standard deviation (SD).

Pain levels were measured using the visual analog scale (VAS), which quantifies subjective pain perception on a scale from 1 to 10, before, during, and at the end of treatment. Changes in pain levels over time were assessed using the Wilcoxon signed-rank test, given the non-parametric nature of the data.

Epithelialization rates were calculated by photographic image analysis. For that purpose, ImageJ software (https://imagej.net/ij/download.html, accessed on 13 October 2024) was used [17]. Camera settings, distance from the wound, and lighting conditions were kept consistent to ensure comparability. Additionally, each image was further calibrated by placing a ruler adjacent to the wound in each case to provide a reference for accurate measurement. This calibration step is crucial for converting pixel measurements to real-world units (e.g., millimeters). Wound edges were manually outlined using the freehand selection tool in ImageJ. This tool allows for precise tracing of the wound margins. Once the wound area was selected, the software calculated the area in square millimeters based on the calibrated scale. The wound area measurements at different time points were recorded and analyzed to determine the reduction in wound size over time. The percentage reduction in wound size was calculated by dividing the decrease in area by the original area and then multiplying by 100. The percentage of wound epithelialization was calculated for each patient and the paired T-test was used to compare the mean reduction in wound size before and after AM treatment. For the patient who did not achieve complete epithelialization, a trend analysis was conducted to assess the percentage reduction in wound size over the course of treatment. All statistical analyses were performed using Prism GraphPad Data Analysis Software Prims (version: 7.0a); *p*-values of less than 0.05 were considered statistically significant.

## 3. Results

This case series includes five patients with chronic wounds lasting more than six weeks despite multiple treatments. The patients’ ages ranged from 61 to 74 years and were referred from the internal medicine unit of the Virgen de la Arrixaca Hospital due the presence of different concomitant pathologies (respiratory, infectious, cardiological, renal, and rheumatological diseases) that related to previous admissions.

### 3.1. Patient’s Profile and Outcomes

**Figure 1:**
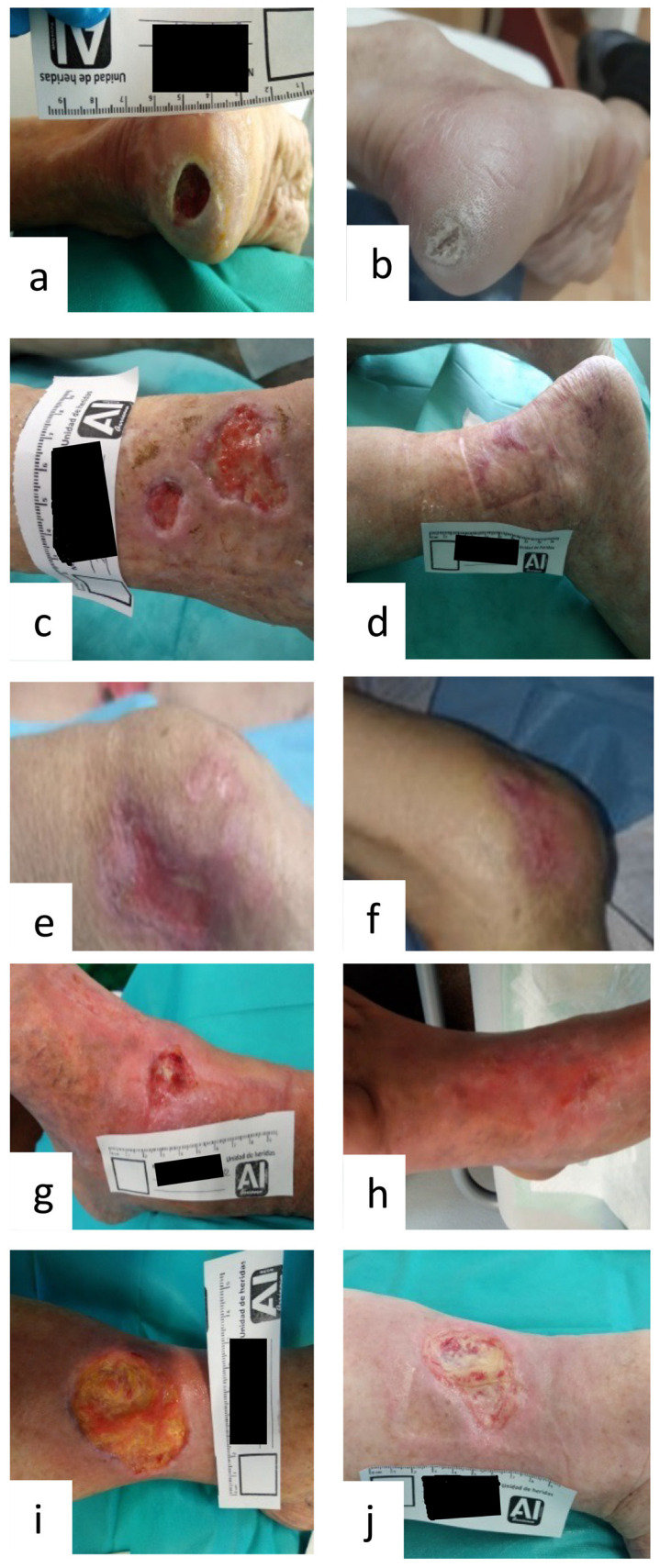
Ulcers treated with amniotic membrane in comorbid patients. (**a**,**c**,**e**,**g**,**i**) Status prior to amniotic membrane treatment; (**b**,**d**,**f**,**h**,**j**) status upon last evaluation. Patient 1 (**a**,**b**), 5 weeks and 5 applications of amniotic membranes. Complete epithelialization. Patient 2 (**c**,**d**), 5 weeks and 5 applications of amniotic membranes. Complete epithelialization. Patient 3 (**e**,**f**), 4 weeks of treatment with AM and 4 applications of amniotic membranes. Complete epithelialization day +85. Patient 4 (**g**,**h**), 6 weeks and 6 applications of amniotic membranes. Complete epithelialization. Patient 5 (**i**,**j**), 14 months and 46 applications of amniotic membranes, showing 40% epithelialization of the wound.

The characteristics of the patients and their wounds are summarized in Table 1. The mean age of the patients was 68.4 ± 5.2 years. Four of the five patients achieved complete epithelialization, while one patient experienced a 40% reduction in wound size, not achieving complete healing before abandoning the procedure due to worsening from concomitant conditions. No treatment-related adverse events were reported, and no clinical signs of infection or tumors were observed in the wound bed or at the edges of the wounds during treatment. Graphic evolution of the wounds, by comparing serial images of each patient before and after the application of MA, helped in assessing the performance of the treatment (Figure 1).

### 3.2. Pain Perception

**Scheme 1:**
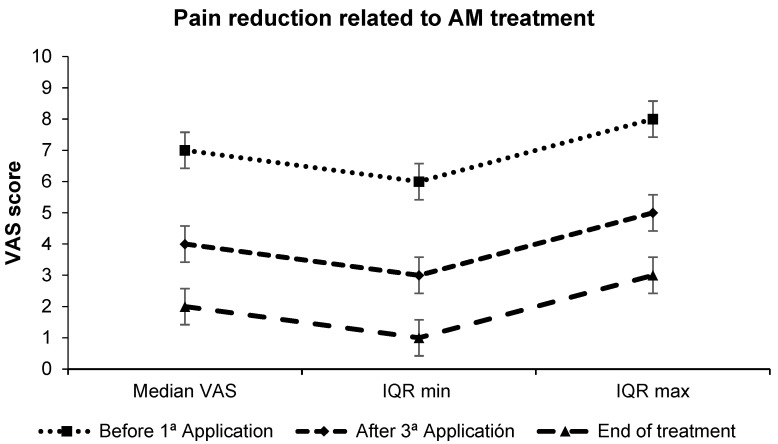
Evolution of pain perception. Error bars show standard error.

Pain levels, measured using the VAS system, decreased significantly over the treatment period. The median VAS score decreased from 7 (IQR 6-8) before the first application to 4 (IQR 3-5) after the third application and to 2 (IQR 1-3) at the end of the treatment (*p* < 0.01, Wilcoxon signed-rank test), as shown in Scheme 1.

### 3.3. Wound Epithelialization

**Scheme 2:**
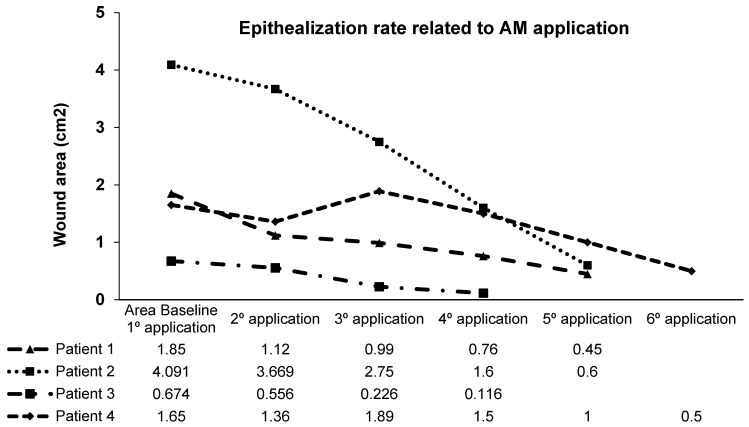
Epithelialization rates achieved during AM treatment until wound closure. Patient 5 data is omitted as this patient did not achieve resolution before abandoning the procedure.

The paired t-test showed a significant reduction in wound size from baseline to the end of the treatment (*p* < 0.01). Four patients achieved 100% epithelialization while one patient achieved just a 40% reduction in wound size after 14 months of treatment. The rate of epithelialization during treatment with AM is shown in Scheme 2.

### 3.4. Microbiology

The microorganisms isolated in the microbiological culture prior to treatment with the amniotic membrane were as follows: two patients had negative cultures (patients 4 and 5); one patient had a polymicrobial flora including *E. coli*, *S. capitis*, *K. oxytoca*, *E. cloacae* complex, *S. epidermidis*, and *S. aureus* (patient 1); one patient had *C. difficile* colitis (patient 3); and one patient had *Enterobacter cloacae* resistant to amoxicillin, ampicillin, and cefuroxime (patient 2).

## 4. Discussion

In this work, we share with the scientific community our expanding experience in the application of AM onto chronic wounds, with a focused series on diabetic foot ulcers in patients suffering from other chronic ailments. In contrast to our previous experiences dealing with traumatic complex chronic wounds [18] and un-comorbid diabetic foot ulcer [14], clinical practice usually reveals that patients commonly associate with a more complex pathological milieu, which is further complicated by the existence of concomitant ailments, not limited to the diabetic condition, and usually related to age. Despite the limited patient studies, the experience shown here confirms the usefulness and safety of AM application for achieving the epithelialization of chronic wounds; even in such a complicated environment, it constitutes a feasible and reliable treatment.

Current research trends in the area of perinatal derivatives show the power of such advanced products for the management and resolution of many degenerative and chronic conditions [19,20]. Several works exist reporting how AM derivatives are useful for facilitating the resolution of different kinds of wounds, including chronic and acute ones [21,22]. A recent study by Horvath et al. [23] confirmed the effectiveness of placenta in wound healing, regardless of its characteristics, which aligns with our findings. In that sense, a recent review [24] highlighted the great healing potency of this kind of treatment. Yet, at the same time, various modifications made to the AM, such as electrospun dressings or hydrogels, have not shown the desired efficacy [24]. Seemingly, in this series, in which a non-altered cryopreserved product was used, the therapy with AM proved beneficial for most of patients (four out of five). Worth noting, based on the data collected, we would recommend a 12-week cut-off point for treatment, in order to avoid unnecessary time and resource expenditure. To this end, we believe that our data could be considered of enhanced value, taking into account that, according to the mentioned review [21], the percentage of studies published in Europe is lower than in other places, likely due to the costly regulation of advanced therapies in place.

Among the benefits of AM treatment for chronic foot and leg ulcers, beyond epithelization and quicker manifestation, we found a reduction in perceived pain by the patients. To our knowledge, reports on pain variation in AM treatment are restricted to the assessment of the analgesic effect of AM reported by Kadkhoda et al. [22] in reducing pain at the palatal donor site after free gingival graft surgery, demonstrating its efficacy, especially in the initial treatment period. As such, our data, although restricted in sample size, constitutes new evidence of the capabilities of AM treatment to improve patients’ quality of life by reducing pain.

The main limitation of this study would be related to the small number of cases constituting this series. However, it should be highlighted that here we reported only on patients with wounds associated with concomitant complex pathologies, rather than more common wounds, such as vascular ulcers. Nonetheless, the conclusions provided by Svobodova et al. [25], regarding the treatment of wounds in polymorbid patients with amniotic membrane versus standard therapy, show similarities with our patients. The low number of cases may also be attributed to the fact that this treatment is currently not available at other hospital centers, and healthcare professionals have not yet been trained in its application, management, and indication. However, efforts are underway to develop a framework within our clinical area allowing the AM therapy knowledge to be distributed to other health centers and to train personnel to apply this therapy, so it would benefit a larger number of patients.

Despite the potency shown, it is evident that, in some cases, this therapy is not effective due to unknown reasons. In this case, the incomplete healing of the wound in patient 5 could be attributed to several factors, including the longer duration of the wound compared to others, bone exposure, the location of the wound in a poorly vascularized area, the patient’s immunosuppression, and possibly the presence of unknown molecular factors. Also, variations in the characteristics of the applied membranes might also play a role. Therefore, in addition to existing studies, molecular analyses of wound exudates before and after AM treatment are needed to identify molecular changes and establish potential research directions.

As a final remark, we want to highlight the effectiveness shown by this treatment, which we find suitable and safe for outpatient application, with potential for significantly improving patients’ quality of life. In that sense, our research group will explore in the near future approaches to enhance the availability of this therapy, with the aim of optimizing its administration for a broader population. Also, as this case series highlights, we need to investigate the molecular differences in the treatment’s mechanism of action between patients who achieve healing and those who do not, in order to identify potential therapeutic targets. Further progress will be shared with the scientific community.

## Figures and Tables

**Table 1:** Patient characteristics and wound treatment with amniotic membrane over time.

Pathologies	Type of Wound/Location	Time of Evolution	Number of AM Applications	Time of Treatment	% Epithelialization
Heart disease, venous insufficiency, arterial hypertension, renal failure, peripheral arterial disease, diabetes mellitus type II (patient 1)	Right heel arterial ulcer	6 months	5	5 weeks	100%
Cryoglobulinemia type I, multiple myeloma (patient 2)	Left foot, Achilles tendon	2 months	5	5 weeks	100%
Calciphylaxis, severe kidney failure (patient 3)	Right knee	12 months	4	4 weeks	100%
Diabetic foot, diabetes mellitus type II (patient 4)	Back of right foot	10 months	6	6 weeks	100%
Fibromyalgia, lymphoproliferative autoimmune syndrome, rhizarthrosis, osteoporosis (patient 5)	Left leg, distal third of the tibia at the dorsal level	4 years	46	14 months	40%

## Data Availability

The data associated with the study were obtained from hospital records.

## Glossary

IQR	Interquartile range, a measure of statistical dispersion. It is calculated as the difference between the third quartile and the first quartile. RQ = Q3 − Q1.
VAS	Visual analogue scale. The visual analog scale is a psychometric response scale that can be used in questionnaires. It is an instrument for measuring subjective characteristics or attitudes. It is numbered from 1 to 10, with 1 being the least pain and 10 being the maximum pain felt.
SOP	standard work protocol.
AEMPS	Spanish Agency of Medicines and Medical Devices
CPU	Cell production unit
